# Exosome-mediated long noncoding RNA (lncRNA) PART1 suppresses malignant progression of oral squamous cell carcinoma via miR-17-5p/SOCS6 axis

**DOI:** 10.55730/1300-0144.5625

**Published:** 2023-01-17

**Authors:** Yuheng DU, Yanjie SHUAI, Hongling WANG, Huisheng LI, Yajing LI

**Affiliations:** 1 Department of Otorhinolaryngology Head and Neck Surgery, Tianjin Medical University Cancer Institute and Hospital, Tianjin, China; 2 Department of First Surgery, Tianjin Public Security Hospital, Tianjin, China

**Keywords:** LncRNA PART1, OSCC, miR-17-5p, SOCS6, exosomes

## Abstract

**Background/aim:**

Exosomes derived from oral squamous cell carcinoma (OSCC) could modulate OSCC development. This study aimed to explore effects of exosome-mediated lncRNA PART1 on OSCC cells.

**Materials and methods:**

This study was performed in Tianjin Medical University Cancer Institute from February 2021 to March 2022. Bioinformatic analysis was performed on the public database GEPIA (http://gepia.cancer-pku.cn/). Exosomes isolated from cell lines squamous cell carcinoma 9 (SCC9) and Centre Antoine Lacassagne-27 (CAL27) were identified by transmission electron microscope and western blot. Exosome-mediated lncRNA PART1, microRNA-17-5p(miR-17-5p) and suppressor of cytokine signaling 6(SOCS6) RNA expressions were assessed by quantitative reverse transcription polymerase chain reaction (RT-qPCR). Cell counting kit-8(CCK-8), caspase-3 activity, and flow cytometry were applied to evaluate OSCC cell viabilities and apoptosis. Meanwhile, OSCC cell migratory ability and invasiveness were evaluated using transwell assay. Bindings between miR-17-5p and lncRNA PART1 or SOCS6 were validated using the luciferase reporter test. Western blot was used for detecting the protein levels of SOCS6, phosphorylated signal transducer and activator of transcription 3 (STAT3) and STAT3.

**Results:**

According to GEPIA, lncRNA PART1 was downregulated in OSCC tissue samples and cells, and it had a positive correlation with the good prognosis of Head and neck squamous cell cancer (HNSCC) patients. After the exosomes from OSCC cells were isolated and verified, PART1 was then confirmed to be secreted by exosomes. Further, overexpression of exosome-mediated lncRNA PART1 inhibited OSCC cell viabilities, migration, and invasiveness but facilitated OSCC cell apoptosis. PART1 upregulated SOCS6 through sponging miR-17-5p. Moreover, exosome-mediated lncRNA PART1 suppressed the phosphorylation of STAT3.

**Conclusion:**

Exosome-mediated lncRNA PART1 could mediate the OSCC progression via miR-17-5p/SOCS6 axis in vitro, suggesting that lncRNA PART1 might be a target for treating OSCC.

## 1. Introduction

Head and neck squamous cell cancer (HNSCC) mainly occurs in the oral cavity, nasal cavity, pharynx, and larynx, and it ranks as the seventh most common cancer [1]. Oral squamous cell carcinoma (OSCC), the main type of HNSCC, is a lethal malignancy with approximately 300,000 new cases per year all over the world [2,3]. Hence, it is of significance to discover new therapeutic targets regarding OSCC.

In recent years, studies have suggested that communication among cells could be achieved through membranes, cell fragments, or extracellular vesicles (EVs) [4]. Exosome, a main subtype of EVs, having abilities in cell-to-cell communication and microenvironment modulation, can be secreted by nearly all kinds of mammalian cells [5]. Moreover, exosome-mediated factors promoted tumorigenesis, drug resistance, metastasis, etc. [6]. Long noncoding RNA (lncRNA) small nucleolar RNA host gene 16(SNHG16), mediated by exosomes, could sponge miR-16-5p to upregulate SMAD family member 5(SMAD5), resulting in cluster of differentiation 73(CD73) upregulation in Vδ1 T cells, promoting the immunosuppressive regulations in breast cancer [7]. Exosome-transmitted lncRNA UFC1 enhanced nonsmall cell lung cancer cell proliferative, migratory, and invasive capacities and decreased cell cycle arrest and apoptosis through suppressing phosphatase and tensin homolog (PTEN) and targeting zeste homolog 2 (EZH2)[8]. As for OSCC, circ_0000199 in exosomes inhibited OSCC cell growth through interplaying with miR-145-5p and miR-29b-3p, which also increased the recurrence rate and mortality of OSCC patients [9]. Exosome-mediated lncRNA APCDD1L-AS1 acted as a competitive endogenous RNA (ceRNA) of miR-1224-5p to elevate nuclear receptor-binding structures of catalytic su(var), enhancer-of-zeste, trithorax (SET) domain protein 2(NSD2), resulting in increased resistance to 5-fluorouracil; it also promoted cell viabilities and inhibited cell apoptosis in OSCC[10]. LncRNA prostatic androgen-regulated transcription 1 (PART1) was transmitted by exosomes and taken by recipient cells, inducing resistance to gefitinib in esophageal squamous cell carcinoma (ESCC)[11]. Moreover, high lncRNA PART1 level has been reported to be linked to good prognosis in patients with tongue squamous cell carcinoma (TSCC) [12,13]. Nevertheless, further regulatory mechanisms of lncRNA PART1 on modulating OSCC cell progression need to be explored.

MicroRNAs (miRNAs), small noncoding RNAs of 19–24nts, are critical in regulating the biological processes of cancer cells through targeting mRNAs [14]. Moreover, based on data of ENCORI (https://starbase.sysu.edu.cn/), binding sites between miR-17a-5p and lncRNA PART1 were predicted, implying underlying interactions between them. Dysregulation of janus kinase-signal transducer and activator of transcription (JAK/STAT) signaling pathway is closely associated with cancer development [15]. Suppressor of cytokine signaling (SOCS) proteins are negative feedback modulators of JAK/STAT signaling pathway, which can prevent excessive cellular responses and maintain cell homeostasis [16]. SOCS6 was targeted by miR-653-5p, facilitating cell proliferation and migration via JAK2/STAT3 signaling pathway in gastric cancer [15]. Beyond that, upregulated SOCS6 has been reported to restrain radio-resistance in ESCC and enhance sensitivity to cisplatin through degradation and ubiquitination of c-Kit[17]. Binding sites between SOCS6 and miR-17a-5p were analyzed using TargetScan (https://www.targetscan.org/vert_72/). Based on our detections, lncRNA PART1, and SOCS6 shared the same binding sites in miR-17-5p. Hence, we hypothesized that LncRNA PART1 modulated OSCC cell progressions via regulating miR-17-5p and SOCS6 axis.

## 2. Materials and methods

### 2.1. Cell culture

Human tongue squamous cell carcinoma cell lines (SCC9 and CAL27) and human normal oral epithelial cells (HOECs) were obtained from American type culture collection (ATCC, USA) and Procell (Wuhan, China), respectively. Human embryonic kidney 293(HEK-293T) cells were purchased from Procell, which were used in the luciferase reporter analysis. Thereafter, Roswell Park Memorial Institute 1640(RPMI-1640, Gibco, USA) supplemented with 10% FBS (Gibco) and 1% penicillin/streptomycin was applied for incubating cells.

### 2.2. Cell transfection

MiR-17-5p mimics/inhibitor and negative controls (NC mimics/inhibitor) were bought from GenePharma (Shanghai, China). LncRNA PART1 overexpressed plasmid (oePART1) and its control (oeNC) were synthesized using lentivirus vectors (GeneChem, Shanghai, China). Meanwhile, SCC9 and CAL27 cells (1 × 10^6^ cells/well) were incubated in 6-well plates and transfection was conducted when the confluence reached 85% using the Lipofectamine 3000 (Invitrogen, USA). Then, 50 pmol miR-17-5p mimics/inhibitor and negative controls respectively were used in the transfection assays. RNA expressions were then examined 24 h after transfection.

### 2.3. Exosomes isolation

Exosomes in SCC9 and CAL27 cells were isolated using the exoEasy Maxi Kit (Qiagen, Germany). First, cell culture was centrifuged for 20 min at 3000 × g to remove cells and cellular debris. Next, the supernatant was filtered to remove particles over 0.8 μm followed by mixing with Buffer XBP. Thereafter, the mixture was bound to exoEasy membrane affinity spin column. Extracted exosomes were rinsed by Buffer XWP and then washed by Buffer XE. Afterwards, exoRNeasy Midi and Maxi Kit(Qiagen) was applied for extracting RNAs.

### 2.4. Transmission electron microscopy

Exosomes were resuspended and fixed by 30 μL 2% paraformaldehyde. Afterwards, glow-discharged copper grid was used to absorb exosomes. Later, glow-discharged copper grids were fixed using 3% glutaraldehyde and then stained by uranyl acetate. Finally, a transmission electron microscope (JEM-1400, JEOL, Japan)was applied for observing exosomes at 100,000 x.

### 2.5. RT-qPCR

Trizol (Invitrogen, USA) was used for segregating total RNA followed by reverse transcription of lncRNA PART1 and SOCS6 with BeyoRT™II Kit (Beyotime, Shanghai, China). Reverse transcription of miR-17a-5p was conducted using Mir-X Kit (Takara, Japan). Then, 7300Plus Real-Time PCR System (Applied Biosystems, USA) with SYBR Green (Applied Biosystem) was applied for RT-qPCR: predenaturation, 95 °C, 30 s and (denaturation, 95 °C, 5 s, annealing, 60 °C, 10 s and extension, 72 °C, 30 s) × 35 cycles. Primers were listed as below, which were: lncRNA PART1 5′-CAATAAGGCAGAAGAAGGTG-3′ (forward), 5′-GGAGAATCTGAAGTCCCAAG-3′ (reverse)[18]; miR-17-5p 5′-CGGCGGCAAAGTGCTTACAG-3′ (forward), 5′-GTGCAGGGTCCGAGGT-3′ (reverse)[19]; SOCS6 5′-GGAATTCATGAAGAAAATCAGTCTGAA-3′ (forward), 5′-CGGAATTCTCAGTAGTGCTTCTCCTGCA-3′ (reverse)[20]; GAPDH 5′-CCTGCCTCTACTGGCGCTGC-3′ (forward), 5′-GCAGTGGGGACACGGAAGGC-3′ (reverse) and U6 5′-CTCGCTTCGGCAGCACA-3′ (forward), 5′-AACGCTTCACGAATTTGCGT-3′ (reverse). GAPDH and U6 were used for normalization of lncRNA PART1, SOCS6, and miR-17-5p, respectively and the relative expression levels were analyzed using 2^-ΔΔCt^ method.

### 2.6. Cell viability detection

Transfected SCC9 and CAL27 cells (1 × 10^4^ cells/well) were seeded in 96-well plates and 10 μL Cell counting kit 8(CCK-8, Beyotime, China) was added at 24 h, 48 h, and 72 h. Thereafter, cells were kept culturing for another 1 h. Optical density (OD) values at 450 nm were observed using a lab microplate reader.

### 2.7. Caspase-3 activity detection

Based on the manufacturer’s protocols, a caspase-3 activity assay kit (Beyotime) was applied for evaluating caspase-3 activities in SCC9 and CAL27 cells. Cells were incubated in 96-well plates. Thereafter, detection buffer and Ac-DEVD-pNA (2 mM) were added to the cell culture and the mixture was incubated for 2 h at 37 °C. The absorbance of caspase-3 was measured at 405 nm using a lab microplate reader.

### 2.8. Flow cytometry

Apoptosis of SCC9 and CAL27 cells was evaluated using an Annexin V-FITC assay kit (Beyotime). First, cells after exosome treatment and transfection were collected and resuspended by Annexin V-FITC binding buffer. Then, cells were mixed with 5 μL Annexin V-FITC and 10 μL PI followed by incubation in darkness for 20 min at 23 °C. Finally, Accuri C6 Plus flow cytometer (BD Biosciences, USA) was used for detecting apoptosis of SCC9 and CAL27 cells.

### 2.9. Transwell assay

SCC9 and CAL27 cells were seeded into upper chambers (Corning, USA) with serum-free medium. Meanwhile, the lower component was added with 600 μL RPMI-1640 medium with 10% FBS. Matrigels (BD Bioscience, USA) were used for invasion detection. After 24 h, cells in the lower component were stained using crystal violet (Beyotime) and numbers of migrated or invaded cells were checked through a microscope.

### 2.10. Luciferase reporter assay

According to data of ENCORI (https://starbase.sysu.edu.cn/) and TargetScan (https://www.targetscan.org/vert_72/), binding sequences of lncRNA PART1 and SOCS6 containing miR-17-5p binding sites and their mutated types were cloned into pmirGLO vector (Promega, USA) to create lncRNA PART1-wt/mut and SOCS6-wt/mut. Thereafter, these vectors were cotransfected into HEK-293T cells with NC mimics or miR-17-5p mimics. Fluorescence was examined using the Dual-Luciferase Reporter Assay System (Promega).

### 2.11. Western blot

RIPA buffer (Beyotime) was applied for lysing SCC9 and CAL27 cells to isolate total protein. Afterwards, proteins were isolated using 10% SDS-PAGE followed by shifting onto PVDF membranes. Then, 5% skim milk was applied for blocking and primary antibodies were used for incubating membranes overnight at 4 °C. Primary antibodies were listed: anti-CD63 (1:1000, ab134045, Abcam, UK), anti-CD9 (1:1000, ab236630), anti-TSG101(1:1000, ab133586), anti-Calnexin (1:1000, ab133615), anti-p-STAT3 (1:1000, ab76315), anti-STAT3 (1:1000, ab68153), and anti-GAPDH (1:1000, ab9484). Thereafter, membranes were cultivated with goat anti-mouse IgG (HRP) (1:2000, ab6789) for 1 h at 23 °C. BeyoECL Moon (Beyotime) was used for developing and Image J (NIH, USA) was for analyzing bands.

### 2.12. Bioinformatics tools

Based on GEPIA(http://gepia.cancer-pku.cn/), the boxplot about lncRNA PART1 expressions in HNSCC was generated. Moreover, the survival analysis correlated with lncRNA PART1 expression in HNSCC patients was performed using the Kaplan-Meier method on GEPIA database. All the clinical data were from the public database GEPIA.

### 2.13. Statistical analysis

GraphPad Prism 9 (GraphPad, USA) and SPSS 19.0 (USA) were utilized to analyze statistical data. If the data distribution fit a normal distribution with equal variance, differences between the two groups were examined using the student’s t-test. One-way ANOVA was applied for analyzing differences between two groups while two-way ANOVA was carried out to analyze data in the CCK-8 assay. Dunnett’s T3 test was applied for adjusting p values in multiple tests. P < 0.032 was defined to be statistically meaningful.

## 3. Results

### 3.1. LncRNA PART1 was downregulated in OSCC tissues and cells

**Figure 1:**
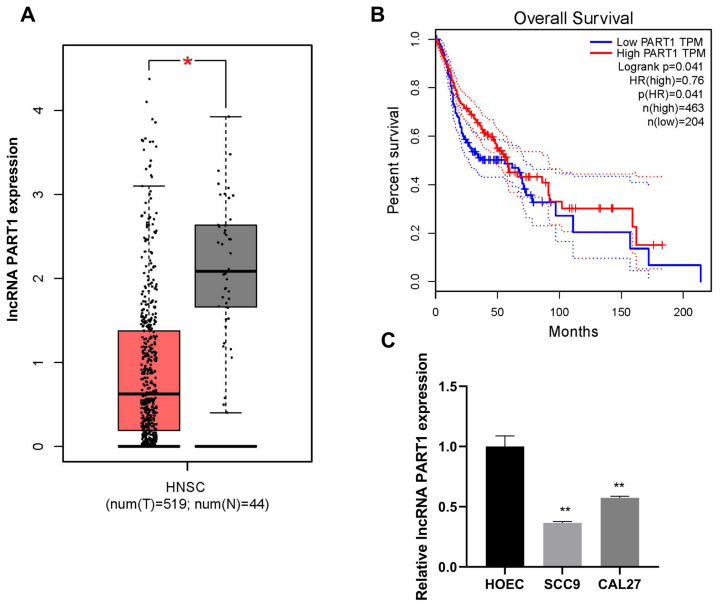
LncRNA PART1 was suppressed in OSCC A: Public database GEPIA (http://gepia.cancer-pku.cn/). Displayed lncRNA PART1 expression in HNSCC tissue samples. B: Kaplan-Meier analysis on GEPIA C: RT-qPCR examined lncRNA PART1 expressions in OSCC cells and HOECs. *p =< 0.032, **p =< 0.0021, ***p =< 0.0002, and ****p < 0.0001.

To explore the effects of lncRNA PART1 on OSCC, we first analyzed its expressions in HNSCC tissues. Based on data of GEPIA (http://gepia.cancer-pku.cn/), lncRNA PART1 expressions were lower in HNSCC tissues (Figure 1A). Kaplan-Meier analysis based on GEPIA database indicated that OSCC patients with higher lncRNA PART1 expression had better prognosis (Figure 1B). We also examined lncRNA PART1 expressions in OSCC cells, which was downregulated in SCC9 and CAL27 cells compared with human oral epithelial cells (Figure 1C). These results suggested that lncRNA PART1 might play a critical role in OSCC.

### 3.2. LncRNA PART1 was transmitted extracellularly via exosomes

**Figure 2:**
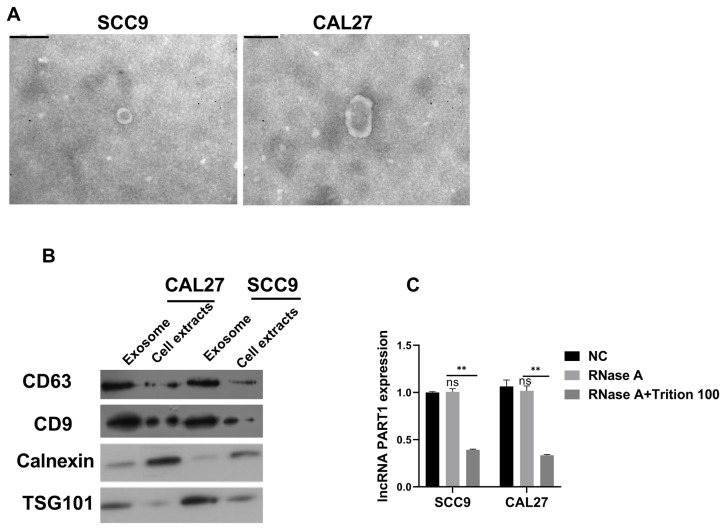
LncRNA PART1 was transmitted extracellularly via exosomes A: TEM examined exosomes at 100,000X. The scale bar = 200nm. B: Western blot evaluated CD63, CD9, TSG101, and Calnexin protein expression. C: RT-qPCR validated lncRNA PART1 expressions in exosomes. *p =< 0.032, **p =< 0.0021, ***p =< 0.0002, and ****p < 0.0001.

Exosomes can be secreted by cells actively, and RNAs in exosomes can be transmitted. To investigate whether lncRNA PART1 was packaged and transferred by exosomes, we extracted exosomes from mediums and observed them using TEM, which showed round and oval membranes and the diameter was 100–150 nm (Figure 2A). Then, exosomes indicators, CD63, CD9 and TSG101, and Calnexin, a negative indicator for exosomes were examined and results showed that their protein expressions of CD63, CD9, and TSG101 were higher in exosomes than cell extracts while Calnexin was low in exosomes, confirming the presence of exosomes (Figure 2B). Furthermore, no significant difference in lncRNA PART1 levels in exosomes was observed after RNase treatment, while RNase and Triton X-10 treatment downregulated lncRNA PART1 expressions, suggesting that lncRNA PART1 was encapsulated by exosomes (Figure 2C).

### 3.3. Overexpression of exosome-mediated lncRNA PART1 inhibited OSCC cell progression

**Figure 3:**
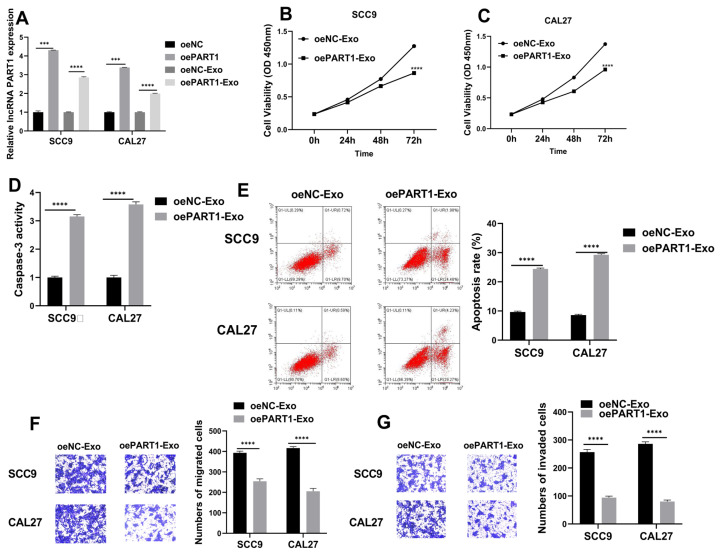
Overexpression of exosome-mediated lncRNA PART1 inhibited OSCC cell progression A: RT-qPCR evaluated lncRNA PART1 levels. B, C: CCK-8 detected viabilities of SCC9 and CAL27 cells after treated with oePART1-Exo. D: Caspase-3 activity detection. E: Flow cytometry evaluated SCC9 and CAL27 cell apoptosis. F, G: Migratory and invasive capacities of SCC9 and CAL27 cells were validated using transwell. *p =< 0.032, **p =< 0.0021, ***p =< 0.0002, and **** p < 0.0001.

Cells transfected with oe-PART1, or its negative control, and respective exosomes extracted from the cell medium were examined for lncRNA PART1 expression. RT-qPCR results indicated that lncRNA PART1 expression was not only upregulated in cells transfected with oe-PART1, but also increased in exosomes extracted from the oe-PART1 cell group (oePART1-Exo) (Figure 3A). This confirmed that lncRNA PART1 is mainly presented in exosomes. Furthermore, CCK8 results revealed that exosomes from SCC9 and CAL27 cells with PART1 upregulation suppressed viabilities (Figures 3B–3C). In contrast, caspase-3 activity and apoptosis rates in SCC9 and CAL27 cells were increased in cells cultured with respective oePART1-Exo (Figures 3D–3E). Additionally, migratory and invasive capacities of OSCC were inhibited by oePART1-Exo (Figures 3F–3G).

### 3.4. MiR-17-5p was sponged by lncRNA PART1

**Figure 4:**
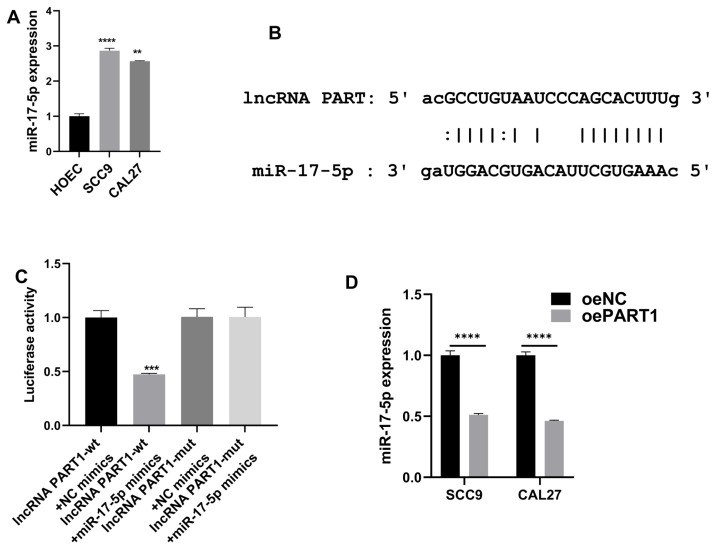
MiR-17-5p was sponged by lncRNA PART1 A: MiR-17-5p expressions in OSCC cells and HOEC were assessed by RT-qPCR. B: ENCORI predicted the bindings between miR-17-5p and lncRNA PART1. C: Luciferase reporter test. D: MiR-17-5p expression in SCC9 and CAL27 cells with lncRNA PART1 upregulation was examined by RT-qPCR. *p =< 0.032, **p =< 0.0021, ***p =< 0.0002, and **** p < 0.0001.

MiR-17-5p expression levels were higher in CAL27 and SCC9 cells than in human oral epithelial cells (Figure 4A). To further reveal the regulatory mechanism of lncRNA PART1 in OSCC cells, we used ENCORI (https://starbase.sysu.edu.cn/) to predict the bindings with miR-17-5p (Figure 4B). Thereafter, a luciferase reporter test was applied, revealing a lower luciferase activity in cells transfected with lncRNA PART1-wt and miR-17-5p mimics (Figure 4C). Furthermore, miR-17-5p expression was decreased with lncRNA PART1 overexpression in cells (Figure 4D). These suggested that miR-17-5p was sponged by lncRNA PART1.

### 3.5. LncRNA PART1 regulated miR-17-5p/SOCS6 to inactivate STAT3 signaling pathway

**Figure 5:**
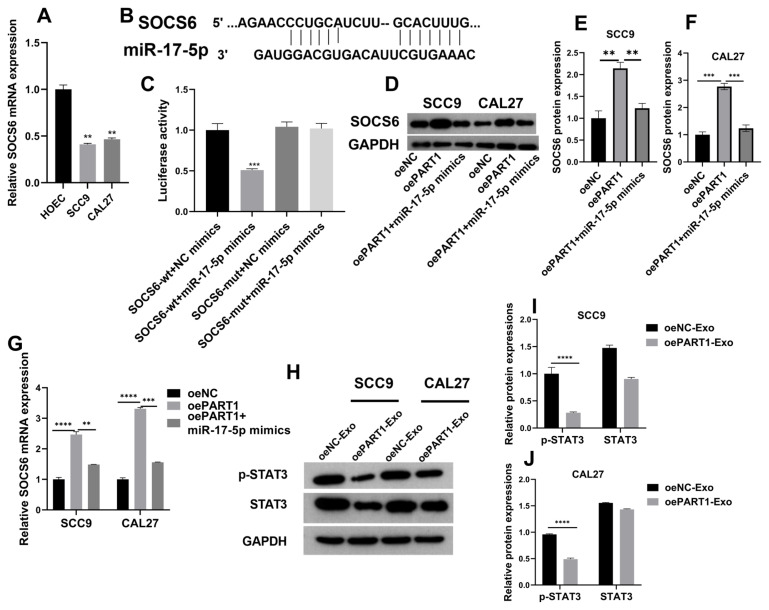
LncRNA PART1 regulated miR-17-5p/SOCS6 to inactivate STAT3 signaling pathway A: SOCS6 mRNA expression in OSCC cells and HOECs were analyzed using RT-qPCR. B: TargetScan provided binding sites between miR-17-5p and SOCS6. C: Luciferase reporter test. D–F: Western blot assays were performed to measure the changes in SOCS6 protein expression. G. SOCS6 mRNA expression in SCC9 and CAL27 cells with PART1 overexpression and additional upregulation of miR-17-5p. H–J: Phosphorylated STAT3 and STAT3 proteins were validated by western blot. *p =< 0.032, **p =< 0.0021, ***p =< 0.0002, and ****p < 0.0001.

Furthermore, SOCS6 mRNA expressions were examined, showing that its expressions were lower in SCC9 and CAL27 cells than in human oral epithelial cells (Figure 5A). Thereafter, we analyzed underlying bindings within miR-17-5p and SOCS6 in homo sapiens using TargetScan (https://www.targetscan.org/vert_72/) (Figure 5B). Further, the luciferase activity turned out the lowest in cells transfected with SOCS6-wt and miR-17-5p mimics (Figure 5C). Later, interactions among lncRNA PART1, miR-17-5p, and SOCS6 were evaluated. SOCS6 protein and mRNA expression were elevated by lncRNA PART1 overexpression, which was partly reversed by miR-17-5p upregulation in cells (Figures 5D–5G). Additionally, the phosphorylation levels of STAT3 in SCC9 and CAL27 cells were also inhibited by oePART1-Exo in OSCC cells (Figures 5H–5J). Hence, lncRNA PART1 might modulate OSCC progression via miR-17-5p/SOCS6 axis by inactivating STAT3 pathway in vitro.

## 4. Discussion

Cancer cells usually produce more exosomes than normal cells and those exosomes provide critical signals for reprogramming stromal cells and cell architectures in tumor microenvironment [21]. Recently, lncRNAs have been reported to be packaged into exosomes to participate in progression of cancers [22]. Exosome-mediated lncRNA ADAMTS9-AS2 restrained cancer cell proliferation, mobility, and EMTby suppressing AKT signaling pathway in OSCC [23]. Exosome-mediated lncRNA LBX1-AS1 sponged miR-182-5p to upregulate FOXO3, leading to inhibited cell proliferation and migration, which also restrained tumor growth in vivo in OSCC[24]. LncRNA PART1 has been demonstrated to be downregulated in tongue squamous cell carcinoma tissues and cells, which could inhibit cell proliferation, invasiveness, and migration via sponging miR-503-5p [13]. Moreover, lncRNA PART1 has also been reported to be suppressed in nasopharyngeal carcinoma cells [25]. In this study, lncRNA PART1 expression was decreased in OSCC tissue samples and cells. Moreover, higher lncRNA PART1 expression was linked to a higher survival rate in HNSC patients. These suggested that lncRNA PART1 might be a tumor suppressor of OSCC. Results from our research unveiled that lncRNA PART1 was transmitted by exosomes derived from OSCC. Furthermore, exosomes from PART1-overexpressing OSCC cells suppressed cell viabilities and migration, and invasiveness but promoted cell apoptosis. Using the ENCORI, the interaction between miR-17-5p and lncRNA PART1 was predicted. Moreover, the luciferase reporter test showed that lncRNA PART1 sponged miR-17-5p directly. Additionally, miR-17-5p expression was upregulated in OSCC cells, which was decreased by lncRNA PART1 overexpression. MiR-17-5p has been reported to be upregulated in HNSC tissues and caused a bad prognosis in HNSC patients, which also accelerated HNSC cell progression via targeting cyclin G2 [26]. Therefore, these results suggested that lncRNA PART1 might sponge miR-17-5p to restrain OSCC cell progression. Moreover, lncRNA PART1 and SOCS6 shared the same binding sites in miR-17-5p. Based on previous studies, lncRNA-miRNA-mRNA ceRNA networks functioned in various cancers [27]. LncRNA RC3H2 acted as a ceRNA sponging miR-101-3p to elevate EZH2, causing the promotion of malignant behavior of OSCC cells [28]. LncRNA TUG1 upregulated distal-less homeobox 1 via competitively sponging miR-524-5p, resulting in facilitated OSCC cell proliferative and migratory abilities [29]. In this study, SOCS6 was promoted by lncRNA PART1 through sponging miR-17-5p, revealing a ceRNA network among these three. As a JAK/STAT signaling pathway suppressor, SOCS6 could cause the inactivation of JAK/STAT signaling pathway [15]. Evidence has verified that increased activation of STAT3 has associated with as many as 50% of all human tumors [30]. Overexpression of EZH2 accelerated STAT3 phosphorylation at pY705, leading to tumor glycolysis, invasion, migration, and epithelial-mesenchymal transition in OSCC [31]. Suppression of phosphorylated STAT3 after icaritin treatment could curb OSCC development by suppressing cell proliferation and promoting cell apoptosis and autophagy [32]. In our study, oePART1-Exo downregulated phosphorylated STAT3 protein expressions in OSCC cells, suggesting that lncRNA PART1 might restrain OSCC malignant behavior via suppressing STAT3 signaling. Taken together, exosome-derived lncRNA PART1 might hamper OSCC progression via miR-17-5p/SOCS6/STAT3 signaling in vitro.
